# Experiences and Attitudes of People with HIV/AIDS: A Systematic Review of Qualitative Studies

**DOI:** 10.3390/ijerph17020639

**Published:** 2020-01-19

**Authors:** Tomás Arias-Colmenero, Mª Ángeles Pérez-Morente, Antonio Jesús Ramos-Morcillo, Concepción Capilla-Díaz, María Ruzafa-Martínez, César Hueso-Montoro

**Affiliations:** 1 Guadalajara Hospital, 19002 Guadalajara, Spain; tac00003@red.ujaen.es; 2 Faculty of Health Sciences, University of Jaén, 23071 Jaén, Spain; 3 Department of Nursing, Faculty of Nursing, University of Murcia, 30100 Espinardo, Spain; ajramos@um.es (A.J.R.-M.); maruzafa@um.es (M.R.-M.); 4 Faculty of Health Sciences, University of Granada, 51001 Ceuta, Spain; conchicd@ugr.es (C.C.-D.); cesarhueso@ugr.es (C.H.-M.)

**Keywords:** HIV/AIDS, systematic review, qualitative approaches

## Abstract

The aim of this article was to explore the experiences and attitudes of people with HIV/AIDS. A systematic review of qualitative studies was carried out. Twenty-seven articles were included, with sample sizes ranging from 3 to 78. Articles from North America, South America, Central America, Europe, and Africa were included. Five topics emerged from the synthesis: feelings about the diagnosis of HIV/AIDS; stigma and HIV/AIDS; changes in sexual behavior after becoming infected; living with the virus; and pregnancy and motherhood in seropositive women. The moment of diagnosis is of vital importance for these people due to feelings such as disappointment, sadness, fear, despair, lack of awareness, and pain. Social support is highly valued among these people and is linked to an improvement in these peoples’ quality of life. Different kinds of stigma accompany people with HIV/AIDS throughout their life, like social stigma, self-stigma, and health professionals’ stigma. Seropositive women who decide to become mothers can feel frustration because they cannot breastfeed. Spirituality helps some people to deal with the fact of being a virus or disease carrier.

## 1. Introduction

HIV is one of the main problems with regard to public health, with greater representation in developing countries [1]. The most affected region is Africa, where almost two thirds of new HIV infections can be found [2]. Worldwide, amongst the population with HIV, 54% of adults and 43% of children are currently being treated with antiretroviral therapy, with the global coverage of these medications for pregnant women or for women who are breastfeeding being approximately 75%.

Being seropositive or having the disease tends to occur in several stages, among which are: the stage of diagnosis, where the person is normally in shock; and the stage of acceptance (positive adaptation) or denial (negative adaptation) [3]. From the beginning of the disease, when it was labelled the so-called “gay-syndrome”, it was characterized by a huge burden of discrimination [4]. This stigma is a negative element that limits the individual’s adaptation to the disease/seropositivity, as well as complicating the management and treatment of the disease; it also creates difficulties in the relationship with the population in general, and with health care professionals [5].

Currently, there are global proposals, such as the well-known 90–90–90, the Joint United Nations Program on HIV/AIDS [6], whose aims include raising awareness of the HIV/AIDS epidemic. This proposal, through an ambitious project, suggests that, by 2020, 90% of seropositive people must be diagnosed in the world, 90% of them must be treated, and 90% of them must be free of viral load. It also suggests that different governments and policies join forces to control the disease. In this sense, this research aims to explore the disease from the individual’s own perspective and, therefore, to contribute to making the daily life of people who suffer from it visible.

The purpose of this review was to explore the experiences and attitudes of people with HIV/AIDS. We consider that knowledge-based outcomes from this study can help improve decision-making on health strategies to cope with HIV, and also guide future research on the topic.

## 2. Materials and Methods

A systematic review of qualitative studies was developed. Our research included studies published in Spanish, Portuguese and English. We selected original articles oriented to qualitative methodologies, whose interest of study was to explore the perspective of people with HIV/AIDS. Studies regarding a pediatric population or focused on adolescence were excluded. We intended to show a broad view regarding the phenomenon under study, which incorporated works from a wide geographical context. That is why different search sources and databases were used, such as CINAHL, PubMed, Lilacs, Cuiden and Google Scholar. In the same way, descriptors from MESH, CINAHL/MeSH, Subject Headings and DeCS (for the Spanish Language) were employed, in addition to non-standardized terms. The English terms that we used were: Human Immunodeficiency Virus, HIV, AIDS, qualitative research or studies; whereas the Spanish terms were: VIH, SIDA, cualitativo. The search was conducted from January to March 2019, including publications until 2018. The oldest article included in the review was published in 2004.

***Search.*** Different search strings were designed with thematic, main, and free descriptors for the different databases. For instance, we used the following search string for the PubMed database: <(HIV[mj] OR Human Immunodeficiency Virus*[tiab] OR Human T Cell Lymphotropic Virus Type III[tiab] OR Human T Cell Leukemia Virus Type III[tiab] OR Lymphadenopathy Associated Virus*[tiab] OR Lymphadenopathy-Associated Viruses[tiab] OR Human T Lymphotropic Virus Type III[tiab] OR AIDS Virus*[tiab] OR Acquired Immunodeficiency Syndrome Virus[tiab]) AND (qualitative research[mh])>. Supplementary File 1 shows the different search strings employed in each data source.

**Figure 1:**
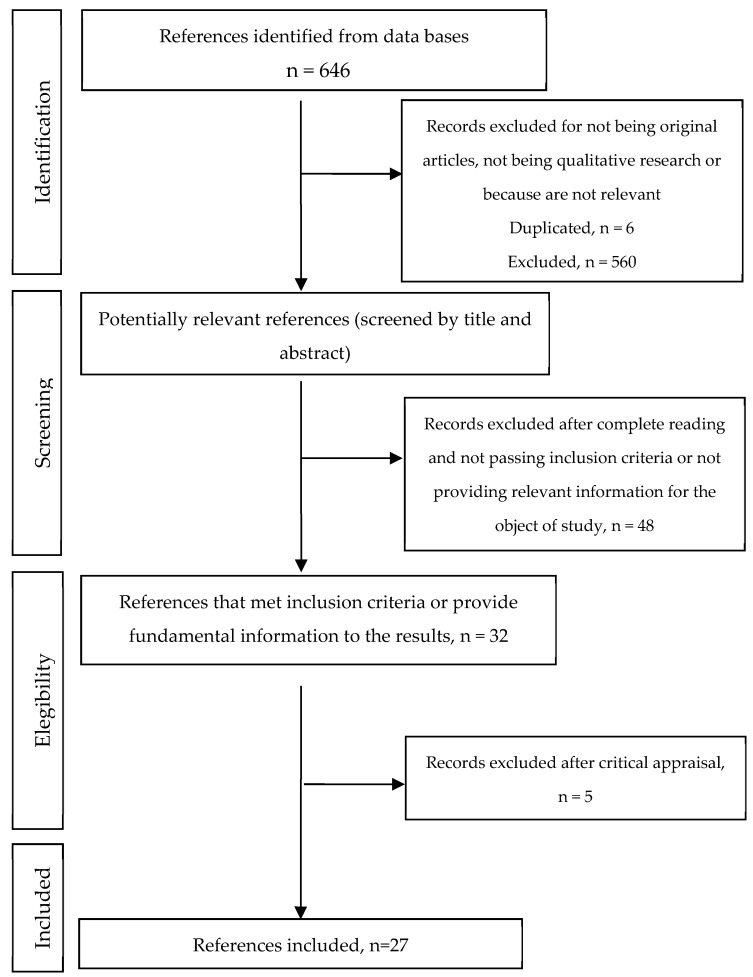
Flow chart of study inclusion and exclusion.

Initially, duplicate studies were excluded and after that, a screening process took place based on: (1) title; (2) abstract; and (3) the full text. The discrepancies regarding article selection were solved by consensus. Finally, the articles’ methodological quality was evaluated. Hence, 27 articles remained in this review. Figure 1 shows the flowchart.

***Critical Evaluation.*** The articles which we considered to be relevant after reading the full text were evaluated via peer review. An evaluation of these articles’ methodological quality was conducted through the CASPe program for qualitative research [7]. The items included in this guide are “present”, “doubtful” and “not on record”. Therefore, some eligibility criteria were proposed; first, that one of the elimination items does not have “not on record” (1, 2, 3), and also that the rest of the items do not have four or more “doubtful” or “not on record” (4, 5, 6, 7, 8, 9). Item number 10 was not evaluated because it does not focus on the applicability of this research in concrete situations, which exceeds the discoveries’ methodological evaluation and evaluation of relevance. The results of this phase are shown in Supplementary File 2.

Despite the fact that 26 articles passed the quality evaluation according to the proposed criteria, we decided to include an article that did not pass it because of the relevance of its results in relation to the aim of the research. On this matter, regarding the syntheses procedures of qualitative studies was followed, which suggests giving priority to the discoveries’ quality in the article selection [8]. Hence, the final number of articles included in the systematic review was 27.

***Data Extraction.*** Relevant data (author/s; participants’ country, number and other features: man or woman -mothers or pregnant-; type of research; qualitative approach chosen in the research and field where it was developed, understanding “Hospital” as any hospital field and “Community” as any association, advice or monitoring clinic or health center) were extracted by the main author of this review and verified by the rest of the authors.

***Data Analysis.*** After repeated reading of the articles, we carried out the narrative synthesis. It consisted of joining the information by means of common topics, creating in this way different categories and subcategories when necessary. The results are presented here, taking the identified topics as the core idea, describing the main discoveries of the different studies in an integrated manner and incorporating, in some discoveries, direct quote of the studies’ informant participants to show evidence of their narratives.

This research is consistent with the guide “Enhancing Transparency in Reporting the Synthesis of Qualitative Research” [9] with the purpose of giving uniformity to the publications of qualitative synthesis studies.

## 3. Results

Of the 27 studies that met inclusion criteria, sample sizes ranged from 3 to 78, with participants coming from Canada, Ireland, Spain, Kenya, Malawi, Ghana, Ethiopia, South Africa, Brazil, Chile, Peru and Mexico. Moreover, 63% of the participants came from the community field, whereas 27% belonged to the hospital one. All of the articles used the interview in different forms, and, furthermore, only two of them used focus groups among their methods of data collection (Table 1).

Five topics emerged after the narrative synthesis: feelings about the diagnosis of HIV/AIDS; stigma and HIV/AIDS; changes in sexual behavior after becoming infected; living with the virus; pregnancy and motherhood in seropositive women (Table 2).

### 3.1. Feelings about the Diagnosis of HIV/AIDS

This category was identified in 33.33% of the articles, which includes the feelings that the study participants experienced after the diagnosis of HIV/AIDS, as well as the different attitudes that they took to face the situation. The feelings we can highlight are disappointment, sadness, fear, despair, lack of awareness, and pain. In some cases, these emotions lead to depression, or they might intensify it. Hence, feelings of frustration might appear as well due to not achieving the targets that the subjects have set in their life [16].

After the diagnosis of HIV/AIDS, it was highlighted that people were afraid of being alone, because the lack of awareness about it causes social exclusion toward the people infected. The acceptance of the diagnosis is difficult; however, it depends on the cultural and social traits of the person. In some cases, people opt for submission to the diagnosis and its consequences or to conformism.

In some cases, they accept that the risky practices they have made in their life have resulted in the fact that they are carriers of the virus and they accept their mistake. One participant of the research by Carrasco et al. [15] expresses it in this way: “...when I was informed that I suffered from HIV, I felt an extremely huge sorrow, an extremely huge helplessness, but, at the same time, I was very calm because I admitted and accepted the mistake that I had made when I did not take care of myself...”. However, it should be noted that there are many ways in which a person could become infected (transmitted from a HIV+ mother during birth, blood transfusion), so it does not follow that each person infected did something wrong, made a mistake. Furthermore, even people felt that they were a mistake, it seems unlikely that every one of them would get to a stage where they all “accept their mistake”.

Many women discover their HIV status (seropositive) in prenatal care or when the children they have had get sick. This leads to an intensification of all of the feelings previously described and, above them, the fear of transmission to their children, in cases where they are pregnant.

After the diagnosis, we can notice in the articles’ results that the advice on and treatment of HIV/AIDS helped participants to accept their situation, avoiding in this way feelings of hopelessness or exclusion. Moreover, they helped to increase their responsibility in regard to self-care, which guarantees longevity and the fact of trying to lead a normal life.

### 3.2. Stigma and HIV/AIDS

This category was identified in 44.44% of the articles, which was, in turn, divided in three subcategories: social stigma, self-stigma and health professionals’ stigma.

#### 3.2.1. Social Stigma

Social stigma is linked to family stigma; that is, the feeling of prejudice against people with HIV/AIDS which, in many cases, results in the social exclusion of the people who suffer from it. Social stigma is a common issue that people infected with the virus suffer, although it is more highlighted in developing countries. For instance, in these countries, women do not undergo a diagnostic test for fear of being judged by people. Therefore, they have to go to other villages to undergo the test because they are afraid of being isolated from the community. Another characteristic of these countries is that women are the ones who undergo these tests, so that men can blame them (even if they are the main carriers) when they are HIV-positive. Seropositive people are still labelled and judged every day, being treated as promiscuous, homosexual or less honorable, which is linked in many cases to the virus being transmitted through sexual contact.

HIV-positive people or people with AIDS make new circles with infected people because they feel free of any judgement. This results in the fact that these people close old social or family circles and they do not divulge the diagnosis. Stigma causes a serologic silence as a means of protection, as well as to avoid discrimination and prejudice. When the serological status is revealed to close people or to relatives, people infected with HIV feel liberated and, generally, accepted. Nevertheless, there are some families that prefer this news to be kept in the privacy of their home to avoid being judged by close people, such as their neighbors. This family acceptance has an influence on the increase in the quality of life, acceptance of the virus/disease and better adherence to antiretroviral therapy in HIV-positive people. In the case of homosexual people with the virus, they frequently suffer the so-called “double stigma”: one because of their sexual orientation, and another because of being seropositive.

#### 3.2.2. Self-Stigma

Most HIV-positive people or people with AIDS, besides suffering discrimination and prejudice by others, also suffer these feelings toward themselves. The fear of transmitting the disease to their relatives or to people in their environment is a common feeling in these people. They even go so far as to take exaggerated hygiene measures or to use different pieces of cutlery to the rest of the family.

They also experience feelings of guilt and embarrassment, as well as the belief that the disease is a divine punishment because of their risky behavior some time ago. In the research by Peñarrieta de Córdova et al. [31], one participant stated that: “...every bad act leads to a bad consequence... It is the price that I am paying because of everything that I have done...”. The feeling of being useless, not respectable to society, and undesirable to other people causes social isolation and the retirement of social circles.

#### 3.2.3. Health Professionals’ Stigma

Some seropositive people take the views of the health professionals who treat them as a reference point because of all the knowledge which they have concerning health. That is why some of these professionals’ practices or attitudes can make people with the virus internalize the discriminatory behaviors that some professionals carry out.

In some cases, the fact that health professionals take additional measures as extra-safety precautions during procedures, when they provide clinical care and treatment, is mentioned. This is obvious from the clinical safety point of view; however, participants might misunderstand it in terms of stigma.

Although the patients reveal that they felt more singled out and judged by these professionals in the past, they still sometimes perceive it in their clinical care. Other results make reference to the opposite. Health professionals support and reinforce people with HIV/AIDS, which leads to a better adherence to the therapy and to the fact that these professionals become people with whom they can relieve their feelings. This support is more emphasized and necessary when people know they are carriers, because of the psychological impact that this entails.

### 3.3. Changes in Sexual Behavior after Becoming Infected

This issue is addressed in 37% of the articles included in this review, so that the main changes, measures and attitudes regarding the sexual behaviors of people with HIV/AIDS are presented. Feelings of anxiety when talking about this matter, insecurity, fears caused by the possible refusal of the others when becoming intimate, decrease in desire and sexual appetite, and apathy and lack of interest are common among seropositive people regarding their sexual lives.

It was highlighted that sexual pleasure and intimacy became affected after diagnosis of HIV/AIDS due to fear of transmitting the virus, guiltiness, and lack of freedom. In the majority of them, a change of behavior after the diagnosis prevails concerning the use of a condom. The goal of its use is to prevent the transmission to their sexual partners or to avoid repeated exposure to the virus. The use of a condom is a limitation for many seropositive people, maybe because of the loss of feeling or freedom of choice as they are “forced” to use them (as a preventive measure). This fact makes the adaptation of the individual to live with HIV difficult.

In some cases, practices like sexual abstinence for fear of infection are reported. Other people deny accepting their seropositivity and they prefer to give up on sex, which even leads to the person’s isolation on several occasions. In the research by Freitas et al. [20], one of the participants expressed: “I cannot be cured, so I stopped going out, I stopped dating, I isolated myself”.

Among these individuals, there is an inability to look for sexual partners with whom they can enjoy life. This is due to the fear of rejection after revealing their serological status, which causes anxiety and constant concern on this matter. On the other hand, the ideal of romantic love and confidence that exists among steady partners (those who are serodiscordant, that is, one of the individuals is carrier of the virus and the other is not) makes them feel less vulnerable to infection themselves, and they forget about the prevention measures.

In developing countries, as can be seen in the study carried out by Sikweyiya et al. [32], men feel a loss of masculinity when they find out about the diagnosis, because they have to use a condom (which is one of the reasons why it is hardly used in these countries) and because they have to reduce the number of their sexual partners (polygamy). Another feeling expressed by men is sadness, which is linked to the impossibility to perpetuate their family name and, therefore, this results in a sense of castration.

Another relevant issue consists of who is in charge of taking care of the prevention means or of accepting unsafe sexual behaviors. As the reviewed studies present, this responsibility can be understood in three different ways. On the one hand, the responsibility lies with the seropositive person, who has the “duty” to protect the others and to take care of themselves. This is the ethical and correct option. On the other hand, the responsibility is shared, that is, both people must decide whether to take precautions or not to avoid risks. Finally, many people defend the idea that the responsibility of looking after and protecting oneself is individual, as is indicated in one of the participants’ statement that appears in the research by Fernández-Dávila et al. [17]: “The boy took it off from me (the condom). I didn’t say anything. Because this depends on him. I do not think it was necessary that he said any word to me...”.

Finally, in spite of understanding concepts like safe sex and preventive measures, condoms are still not used as they should to avoid new infections. As Juárez and Pozo [25] specified in their research, people who are in antiretroviral therapy, despite the fact that the possibilities of infecting the rest have only diminished, feel invulnerable. This makes them relax and employ risky behaviors in their sexual practices.

### 3.4. Living with the Virus

This category was identified in 44.44% of the articles, where the confrontation strategies that people with HIV/AIDS apply in their lives are principally addressed. According to many of the participants of the studies, being seropositive, or a carrier of the disease, means that they increase their self-care, fight for their lives and love other people more in order to receive the necessary support.

To overcome the diagnosis with the desire to continue living requires that people with HIV/AIDS make changes in their lifestyles voluntarily and with the full conviction that they are necessary actions to lead a “normal” life. Understanding how the disease functions and what it involves is fundamental for the participants of the different studies reviewed. What helps to put bad practices aside is to focus on healthy habits such as maintaining a positive attitude, moderate physical exercise, a healthy diet and trying to have an active social life. This helps to avoid depression, loneliness, isolation and hopelessness.

Among the responsibilities that being seropositive entails, we can include taking medication (antiretrovirals), which help these people to retain their wellbeing as the age. Like Juárez and Pozo [25] mentioned in their research, participants who took medication noticed some improvement in their quality of life. That is why adherence to the therapy and good monitoring is important for them. In Oliveira’s article [30], one of the participants states: “It is a responsibility, because you must take that medication, you must have medical monitoring and you must be careful because you can develop some other diseases”.

In developing countries, men who are infected by the virus believe that the search for social support to cope with HIV disease or with being HIV-seropositive is a sign of weakness. Nevertheless, some others express that, after being diagnosed, they had to change their life a lot and to adapt themselves to this new situation. Having to take medication made them feel prisoners and it made the acceptance and adaptation to life with HIV/AIDS difficult.

Social support is valued highly among these people. The desire to have more social relationships in their lives is expressed, because this helps them to overcome the negative situation linked with the virus. Their close family and friends are an essential source of support, helping them to make their everyday life more bearable and to make their adaptation positive. Most seropositive people learn to give more value to life, family and friends, as they already know that they are fundamental pillars of support, just like one participant states in the research by Braga et al. [13]: “In this case, it happens that you give more value to life”. Some others, however, avoid speaking about the disease/seropositivity and the feelings that it entails with people close to them, omitting in this way the problem and showing some maladjustment.

Finally, another way in which participants attempt to confront the diagnosis and to live with the virus is to get close to religion, which emerges as an emotional support. Faith in some superior being fills these people with motivation, relief, self-improvement and strength. They ask for courage through prayer to not to fall into depression. In Neves and Gir researched [29], one participant declares: “I devoted myself to God’s hands, he is going to give me the answer”. However, other people in De la Cruz et al. [16] described that God punished them for something wrong that they had done in spite of being faithful believers, which creates some uncertainty in them. 

### 3.5. Pregnancy and Motherhood in Seropositive Women

This category was identified in 37.03% of the articles. It includes comments of seropositive women, some of them being mothers, some others pregnant, and some others with the intention of having offspring. Several issues, such as the causes of becoming pregnant, breastfeeding, and the feelings they experience regarding pregnancy with their condition of seropositivity, are addressed.

Among the different reasons that women have for continuing with their pregnancy, as most of them are not planned, we found a need to satisfy their spouses or count on their support. On occasion, family is another kind of support that helps them to continue. Nevertheless, some other times they advise them not to continue with the pregnancy to focus on their own health, because of their condition. Some ecclesiastic communities support these women and encourage them during the motherhood process. Lastly, the most important cause in this category is their own feelings and the availability of antiretrovirals. Some women who are diagnosed before pregnancy tend to be more negative about pregnancy due to the concern about vertical transmission.

Many seropositive women should be conscious of the right to motherhood, because they have the same rights as any other women. Many participants of the studies included in the review are aware of this. However, some others, in spite of knowing this, prefer to refrain from motherhood for fear of transmitting the virus, even if it is desired. Regarding breastfeeding, we found distinguishable comments among women who are treated with antiretrovirals and who decide to breastfeed, either by choice or because of the social pressure, and women who, with regret, avoid breastfeeding for the baby’s benefit.

With respect to the first group, in many countries, especially in developing ones, it should be noted that breastfeeding is a cultural norm which continues through generations. Women who are virus carriers live with the difficulty of motherhood in these communities, as Acheampong et al. [10] described, and they normally suffer and feel pressured when they breastfeed the baby. Among the feelings that they experience, we can highlight the fear and dread of transmitting the virus to their children through their milk, anxiety because of the uncertainty of knowing if their children are contaminated or not by drinking their milk, and the feeling of guilt when they contract HIV because the responsibility is theirs alone. Hope for the use of antiretrovirals and the effect that these have when they reduce the burden to almost imperceptible levels have also been shown.

In the second group, we find mothers who present feelings of failure, sorrow, helplessness or suffering. One participant of the study by Sousa and Gimeniz [33] points out: “It was my dream to have a child and to see him nurse... When he was crying, I could breastfeed him and see how he stopped crying”. For the majority, breastfeeding was a symbol of motherhood.

Because of the fact that these women are recommended not to breastfeed, to reduce the virus transmission to their children, the people around them have many prejudices when they see them feeding their children with infant formula. This means that these women do not reveal their diagnosis for fear of rejection, either of themselves or of their children in the future, having to lie on several occasions, as one participant explains in the article by Linder et al. [26]: “When people ask me if I don’t breastfeed, I say that I have an inverted nipple and, although it is true, it is an excuse as well...”. Many of them stated that the information they received from health care providers about why they must not breastfeed was very superficial. 

Finally, motherhood is perceived as a support in their lives, giving seropositive women a reason to continue living eagerly. They have hope of seeing their children grow up and this is a positive factor in the face of the disease/seropositivity. These mothers usually overprotect their children in order to avoid suffering and rejection from people, as was described by Spindola et al. [34] in their research. Motherhood is a positive factor with respect to the adherence to antiretroviral therapy as well, because the possibility of their children growing up healthily encourages mothers to follow health recommendations.

## 4. Discussion

Systematic reviews of qualitative studies provide a broad view of the experiences of people facing health problems. This research focused on analyzing the experiences and attitudes of people who live with HIV/AIDS, based on a wide review that includes works from several countries, with representation in North America, South America, Central America, Europe and Africa. The analysis allowed us to identify the common elements regarding the feelings when facing the seropositivity/disease diagnosis, the stigma, sexual behaviors, and motherhood, which consolidates the work’s international relevance. This review updates the discoveries which were already generated in previous works of similar characteristics, although they were published more than 10 years ago [37,38]. On the other hand, the publication of systematic reviews in this field has progressed in recent years [39,40,41,42,43]; however, the ones that include qualitative research as a source of results or that focus on very specific aspects are scarce. This strengthens the review that is presented in this work, as it is based on qualitative studies, helping topics which were not treated before to emerge.

Based on our results, we can highlight the stigma that people who are carriers of HIV suffer. Three types of stigma (social stigma, self-stigma and health professionals’ stigma) were relevant in the results. In accordance with the research by Sandelowski et al. [38], in their metasynthesis, although they focus on the female population, one of the problems of revealing the diagnosis to other people is the prejudices that exist toward seropositive people. On the one hand, when they reveal it, they feel relief and their relationships now provide authenticity. On the other hand, it can also be a reason for social isolation because of the non-acceptance of the others. Barroso et al. [37], in their metasynthesis, supported the results obtained in the research about social relationships, as these are a foothold to better adapt to the virus or to the disease. On the other hand, Villa et al. [40] confirmed in their literature review that the psychological aspects of seropositive people are reinforced by the social support they receive, which helps their adherence to the therapy and improves their quality of life. This last idea was also shown by Tavera [41] in her systematic review.

Regarding adherence to the therapy, in one review, Puigventós et al. [39] stated that, in general, people with HIV/AIDS adapted well to the therapy. In the cases in which they do not adhere, the reasons might be that they are social outcasts (stigma), that they are minors, or the lack of motivation, among others. In the results obtained, we observed that, for instance, motherhood is a positive factor (apart from social support) in better adherence to the therapy, reducing in this way the possibility of vertical transmission to the baby. Concerning this last matter, guides for HIV/AIDS management have been published [42,44] which are particularly interesting regarding the approach to motherhood, including recommendations which result in a reduction of vertical transmission.

Among the ways to adapt to the disease/seropositivity, we found in the results of this research that religion or the practice of healthy habits favored its normalization in their lives. This is in line with the proposals suggested by other studies [37,41], which claim that spirituality helps some people to confront HIV/AIDS. In addition, it is stated that understanding the disease makes the adaptation favorable as well, as it helps them to carry out positive strategies, such as physical exercise, changes in their diet or safe sexual behaviors. Benito [43], in his systematic review, explained that physical exercise in people with HIV/AIDS makes them gain weight and is favorable to their psychological wellbeing.

Based on the results obtained, we suggest the further exploration of themes like the experiences of pregnant women or of mothers and their sexual behaviors in future research, as the fact that some HIV carriers adapt to their situation and some others deny it, despite knowing the risks, can be underlined. Likewise, new research on the moment of diagnosis would be enriching, as it is a crucial moment because of the great psychological impact that it entails.

This review is not exempt from limitations. First, even though the sources used for the studies’ search are pertinent, they might not give an account of all of the relevant studies for the objective of this research. To compensate for this limitation, it should be pointed out that the database and the base of resources used are specific to the Health Sciences area (the area in which this research is circumscribed), so that they are widely known and relevant sources in this area. Although no exclusion criteria was applied on a geographical basis, there are regions such as Asia that are not represented in this review, which may be of interest in future research. In this regard, it would also be interesting in future research to locate studies published in languages other than those included in this review.

On the other hand, an excessive number of duplicate documents were avoided, which could have happened with the use of other databases that might have a high degree of overlap with the ones used in this review. Another limitation of this review involves the synthesis procedure. We opted for a classic procedure of narrative synthesis, which limits the descriptive and explanatory ability of the studied phenomenon. It would be relevant to progress toward metasynthesis procedures in future research and, in this regard, the discoveries of this review might be the base on which this future research can be oriented.

Finally, it would be interesting to specifically include in future research an analysis that could differentiate the findings of the studies analyzed based on various factors. For example, how people feel about being diagnosed is likely to be qualitatively different when there are few options compared to when people can live long lives with effective treatment. It is important to note how the country of origin is related to such feelings, as those in under-developed countries may have less access to treatment, which could impact their feelings about the diagnosis.

## 5. Conclusions

Most of the people who are carriers of the virus have common feelings when they are informed about their seropositivity. Among them, we found disappointment, sadness, fear, despair, lack of awareness and pain. Sometimes, the diagnosis might lead to depression and social isolation. Social culture and environment are determining factors regarding the acceptance of the diagnosis. Intimacy and sexual pleasure are affected after the disclosure of the diagnosis; some seropositive people feel a decrease of their sexual appetite and, in general, there are changes in their sexual behaviors (e.g. use of condoms). In other cases, they opt for abstinence or, on the contrary, for risky practices, despite knowing their consequences.

In the case of pregnant women, many of them find out about the diagnosis when they get pregnant. Some others decide to have children in spite of being carriers and the causes that drive them to do this might be the satisfaction of being mothers or the need to satisfy their partners. Being mothers is a positive factor to fight HIV/AIDS, because it gives them the strength to continue and see how their children grow up. Furthermore, breastfeeding generates distinguishable comments between the ones who desire it and find themselves forced to breastfeed because of the social pressure, for whom the use of antiretrovirals relieves their fears in the face of transmission danger, and the ones who opt for not breastfeeding, showing an evident helplessness.

The fact of being seropositive implies a high degree of stigma, as the prejudices against people with HIV/AIDS are evident. Some people lean on their intimate social circle to confront the disease or seropositivity. Religious practices are a positive factor in which they take refuge as well.

### Practical Implications

In the moment of diagnosis, the approach of healthcare providers is of vital importance, because of the impact that the news about being carriers causes on people. Thus, enough support and advice must be offered to these people to avoid future isolation and for appropriate therapeutic adherence. In the same way, correct health education is key to avoiding risk behaviors. It is relevant, from the social environment, to ensure the inclusion of these people in society, avoiding social exclusion.

Regarding pregnant women, an early diagnosis of HIV status makes it possible to adopt measures that drastically reduce the risk of mother-to-child transmission. The information provided about the risk of breastfeeding babies must be complete. The fact that these mothers fully assimilate the information before they make a decision must be checked, avoiding uncertainty, sadness or feelings of helplessness.

Finally, the COCHRANE collaboration recognizes that “evidence from qualitative studies that explore the experience of those involved in providing and receiving interventions, and studies evaluating factors that shape the implementation of interventions, have an important role in ensuring that systematic reviews are of maximum value to policy, practice and consumer decision-making” [45]. Therefore, this review offers an understanding of the perceptions and feelings of people with HIV/AIDS, and thus it can help to improve the implementation of interventions focused on people and guide public health policies or the development of protocols and clinical practice guidelines, which are in tune with the UNAIDS proposal worldwide [6].

## Figures and Tables

**Table 1:** Summary of studies included in the review and narrative synthesis.

Authors, Publication Date and Setting	Context	Design	N	Sample Characteristics (Men or Women -Mothers or Pregnant-)	Method of Data Collection	Type of Interview
Acheampong et al., 2017 (Ghana) [10]	Hospital	Descriptive-exploratory	13	Mothers	Semi-structured interviews	Individual
Akhtar et al., 2017 (Canada) [11]	Community	Interpretative	10	Women	Semi-structured interviews	Individual
Biseck, et al., 2015 (Malaui) [12]	Community	Descriptive	35	Pregnant	Semi-structured interviews	Individual
Braga et al., 2016 (Brazil) [13]	Hospital	Social Representations Theory (SRT)	30	Men and women	Semi-structured interviews	Individual
Carlesso et al., 2011 (Brazil) [14]	Community	Descriptive-exploratory	11	Women	Semi-structured interviews	Individual
Carrasco et al., 2013 (Chile) [15]	Hospital	Phenomenological	15	Men and women	In-depth interviews	Individual
De la Cruz et al., 2016 (Canada) [16]	Community	Narrative	3	Men and women	In-depth interviews	Individual
Fernández-Dávila et al., 2013 (Spain) [17]	Community	Grounded Theory	78	Men	Interviews & focus groups	Individual & group
Figueiredo et al., 2015 (Brazil) [18]	Hospital	Descriptive-exploratory	10	Mothers	Semi-structured interviews	Individual
France et al., 2015 (Ireland) [19]	Community	Interpretative	17	Men and women	Semi-structured interviews & written statements	Individual
Freitas et al., 2000 (Brazil) [20]	Community	Descriptive	5	Men and women	Semi-structured interviews	Individual
Freitas et al., 2017 (Brazil) [21]	Community	Descriptive	30	Men and women	Interviews	Individual
French et al., 2015 (South Africa) [22]	Community	Descriptive-interpretative	23	Men and women	Open-ended interview	Individual
Gardner 2013 (Kenya) [23]	Community	Phenomenological	29	Women	Open semi-structured interviews	Individual
Gonçalves et al., 2013 (Brazil) [24]	Community	Descriptive	12	Women	Semi-structured interviews	Individual
Juárez & Pozo 2010 (Peru) [25]	Hospital	Descriptive-exploratory	40	Men and women	In-depth interviews & focus groups	Individual & group
Linder et al., 2016 (Brazil) [26]	Hospital	Descriptive-exploratory	10	Women	Semi-structured interviews	Individual
Lôbo et al., 2012 (Brazil) [27]	Hospital	Descriptive-exploratory	13	Men and women	Semi-structured interviews	Individual
Matos Oliveira et al., 2016 (Brazil) [28]	Community	Descriptive	13	Women	Semi-structured interviews	Individual
Neves & Gir 2006 (Brazil) [29]	Community	Descriptive	14	Mothers	Semi-structured interviews	Individual
Oliveira et al., 2015 (Brazil) [30]	Community	Descriptive	11	Women	Semi-structured interviews	Individual
Peñarrieta de Córdova et al., 2006 (Mexico) [31]	Community	Descriptive	28	Men and women	In-depth interviews	Individual
Sikweviva et al., 2014 (South Africa) [32]	Community	Interpretative	18	Men	In-depth interviews	Individual
Sousa Paiva & Gimeniz Calvão 2004 (Brazil) [33]	Community	Descriptive-exploratory	13	Mothers and pregnant	Semi-structured interviews	Individual
Spindola et al., 2015 (Brazil) [34]	Hospital	Descriptive	10	Pregnant	Semi-structured interviews	Individual
Teixeira et al., 2013 (Brazil) [35]	Hospital	Descriptive-exploratory	8	Women	Semi-structured interviews	Individual
Wodajo et al., 2017 (Ethiopia) [36]	Hospital	Exploratory	16	Men and women	In-depth interviews	Individual

Source: own elaboration.

**Table 2:** Categories identified in each study.

	*Feelings about the Diagnosis of HIV/AIDS*	*Stigma and HIV/AIDS*	*Changes in Sexual Behavior after Becoming Infected*	*Living with the Virus*	*Pregnancy and Motherhood in Seropositive Women*
Acheampong et al., 2017 [10]					✓
Akhtar et al., 2017 [11]		✓		✓	
Biseck, et al., 2015 [12]					✓
Braga et al., 2016 [13]	✓			✓	
Carlesso et al., 2011 [14]	✓			✓	
Carrasco et al., 2013 [15]	✓	✓	✓	✓	
De la Cruz et al., 2016 [16]	✓	✓		✓	
Fernández-Dávila et al., 2013 [17]			✓		
Figueiredo et al., 2015 [18]	✓		✓	✓	✓
France et al., 2015 [19]		✓	✓		
Freitas et al., 2000 [20]			✓		
Freitas et al., 2017 [21]		✓		✓	
French et al., 2015 [22]		✓			
Gardner 2013 [23]	✓	✓	✓		
Gonçalves et al., 2013 [24]	✓				✓
Juárez & Pozo 2010 [25]			✓	✓	
Linder et al., 2016 [26]					✓
Lôbo et al., 2012 [27]		✓	✓	✓	
Matos Oliveira et al., 2016 [28]					✓
Neves & Gir 2006 [29]			✓	✓	✓
Oliveira et al., 2015 [30]	✓	✓		✓	
Peñarrieta de Córdova et al., 2006 [31]		✓			
Sikweviva et al., 2014 [32]		✓	✓	✓	
Sousa & Gimeniz 2004 [33]					✓
Spindola et al., 2015 [34]	✓				✓
Teixeira et al., 2013 [35]					✓
Wodajo et al., 2017 [36]		✓			

Note: We opted to unify the references by mentioning the first and the second author, if there were two authors. From three authors or more, the first is mentioned and we add “et al.” for the rest. Note 1: “✓” = Article included in the category. Source: own elaboration.

## Supplementary Materials

The following are available online at https://www.mdpi.com/1660-4601/17/2/639/s1, Table S1: Search strategy in the databases; Table S2: Results after the methodological evaluation CASPe.

## References

[1] World Health Organization (2017). 10 Facts On HIV/AIDS. http://origin.who.int/features/factfiles/hiv/en/.

[2] World Health Organization (2018). Key Facts VIH/SIDA. https://www.who.int/news-room/fact-sheets/detail/hiv-aids.

[3] Pérez Fernández P., Gómez Rodríguez M.S., Pinto González L. (2018). Vivencias sentidas hoy por personas que viven con VIH/SIDA. Rev. Ética De Los Cuid..

[4] Castillo J.A.L. (2004). Infección-enfermedad por VIH/SIDA. Medisan.

[5] Steward W.T., Herek G.M., Ramakrishna J., Bharat S., Chandy S., Wrubel J., Ekstrand M.L. (2008). Estigma relacionado con el VIH: Adaptación de un marco teórico para su uso en la india. Soc. Sci. Med..

[6] UNAIDS (2014). 90-90-90: An Ambitious Treatment Target to Help End the AIDS Epidemic.

[7] Cano Arana A., González Gil T., Cabello López J. (2010). Plantilla para ayudarte a entender un estudio cualitativo. Guías CASPe De Lectura Crítica De La Literatura Médica.

[8] Sandelowski M., Barroso J., Voils C.I. (2007). Using qualitative metasummary to synthesize qualitative and quantitative descriptive findings. Res. Nurs. Health.

[9] Tong A., Flemming K., Mcinnes E., Oliver S., Craig J. (2012). Enhancing transparency in reporting the synthesis of qualitative research: ENTREQ. BMC Med. Res. Methodol..

[10] Acheampong A.K., Naab F., Kwashie A. (2017). Qualitative exploration of psychological reactions and coping strategies of breastfeeding mothers living with HIV in the Greater Accra Region of Ghana. Int. Breastfeed. J..

[11] Akhtar N.F., Garcha R.K., Solomon P. (2017). Experiences of women aging with the human immunodeficiency virus: A qualitative study. Can. J. Occup. Ther..

[12] Biseck T., Kumwenda S., Kalulu K., Chidziwisano K., Kalumbi L. (2015). Exploring fertility decisions among pregnant HIV-positive women on antiretroviral therapy at a health centre in balaka, malawi: A descriptive qualitative. Malawi Med. J..

[13] Braga R.M.O., Lima T.P., Gomes A.M.T., Oliveira D.C.D., Spindola T., Marques S.C. (2016). Social representations of HIV/AIDS for people living with the syndrome. Gale Acad. Onefile.

[14] Carlesso A., Cecchetto F.H., Silva E.F.D. (2011). Women infected by the human immunodeficiency virus: Experienced feelings regarding the sickness. J. Nurs. UFPE.

[15] Carrasco P., Araya Gutiérrez A., Loayza Godoy C., Ferrer Lagunas L., Trujillo Guarda C., Fernández Sarmiento A., Pérez Cortés C. (2013). How to Understand the Experience of Persons Living with HIV: Implications for Clinical Practice and Research. Aquichán.

[16] De la Cruz A., Caine V., Mill J. (2016). Sub-Saharan African immigrants living with HIV in Canada: A narrative inquiry. Int. J. Migr. Health Soc. Care.

[17] Fernández-Dávila P., Morales Carmona A. (2013). Discursos sobre la responsabilidad sexual en hombres VIH-positivos que tienen sexo con hombres. Rev. Esp. Salud Publica.

[18] Figueiredo R.M.B., Thomé A., Pinto P.C., Prates C.D.S. (2015). Vivências de mães soropositivas para o HIV acompanhadas no serviço de assistência especializada. Rev. Enferm. UFSM.

[19] France N.F., Mcdonald S.H., Conroy R.R., Byrne E., Mallouris C., Hodgson I., Larkan F.N. (2015). “An unspoken world of unspoken things”: A study identifying and exploring core beliefs underlying self-stigma among people living with HIV and AIDS in Ireland. Swiss Med. Wkly..

[20] Freitas M.R.I., Gir E., Rodrigues A.R.F. (2000). Understanding the sexuality of individuals of HIV-1. Rev. Esc. Enferm. USP.

[21] Freitas M.I.D.F., Bonolo P.D.F., Miranda W.D.D., Guimarães M.D.C. (2017). Interactions and the antiretroviral therapy adherence among people living with HIV/AIDS. Rev. Min. Enferm..

[22] French H., Greeff M., Watson M.J., Doak C.M. (2015). HIV stigma and disclosure experiences of people living with HIV in an urban and a rural setting. AIDS Care.

[23] Gardner J. (2013). The experiences of HIV-positive women living in an African village: Perceptions of voluntary counseling and testing programs. J. Transcult. Nurs..

[24] Gonçalves V.F., Teixeira D.Q., Oliveira P.F.D., Sousa T.H.E. (2013). HIV-seropositive women: Understanding, feelings and experience before motherhood. Rev. Bras. Promoc. Saude.

[25] Juárez-Vílchez J.P., Pozo E.J. (2010). Risk sexual behavior among people living with HIV/AIDS and receiving antiretroviral therapy in Piura, Peru. Rev. Peru. Med. Exp Salud Publica.

[26] Linder V., Chaves S.E., Strapasson M. (2016). Perceptions of living women with human immunodeficiency virus about breastfeeding inability. Enferm. Foco.

[27] Lôbo M.B., Silva S.R.F.F., Santos D.S. (2012). Bedroom secrets: Safe sex knowledge and practice by people living with HIV/AIDS. Rev. Eletr. Enf..

[28] Matos Oliveira G., Aguiar Carvalho M.F.A., Argolo Teixeira M., Cardoso Coelho E.A., Teixeira Araújo R. (2016). Perception of seropositive women for hiv about reproductive rights. J. Nurs. UFPE.

[29] Neves L.A.S., Gir E. (2006). HIV positive mother´s beliefs about mother to child transmission. Rev. Lat.-Am. Enferm..

[30] Oliveira A.D.F., Araújo Vieira M.C., Pinheiro Costa e Silva S., Mistura C., da Silva Jacobi C., de Souza Carvalho e Lira M.O. (2015). Effects of HIV in daily life of women living with AIDS. Rev. Pesqui. Cuid. Fundam. Online.

[31] Peñarrieta de Córdova M.I., Rivera A.M., Piñones Martínez S., Quintero Valle L.M. (2006). Experience of living with AIDS in a Latin country: A qualitative analysis. Cult. Cuid..

[32] Sikweyiya Y.M., Jewkes R., Dunkle K. (2014). Impact of HIV on and the constructions of masculinities among HIV-positive men in south africa: Implications for secondary prevention programs. Glob. Health Action.

[33] Sousa Paiva S., Gimeniz Calvão M.T. (2004). Feelings of pregnant and post-partum women with HIV/AIDS about not breastfeeding. Texto Contexto-Enferm..

[34] Spindola T., Dantas K.T.B., Cadavez N.F.V., Fonte V.R.F.D., Oliveira D.C.D. (2015). Maternity perception by pregnant women living with HIV. Investig. Educ. Enferm..

[35] Teixeira S.V.B., Silva G.S., Silva C.S., Moura M.A.V. (2013). Women living with hiv: The decision to become pregnant. Rev. Pesqui. Cuid. Fundam. Online.

[36] Wodajo B.S., Thupayagale-Tshweneagae G., Akpor O.A. (2017). Stigma and discrimination within the ethiopian health care settings: Views of inpatients living with human immunodeficiency virus and acquired immune deficiency syndrome. Afr. J. Prim. Health Care Fam. Med..

[37] Barroso J., Powell-Cope G.M. (2000). Metasynthesis of qualitative research on living with HIV infection. Qual. Health Res..

[38] Sandelowski M., Lambe C., Barroso J. (2004). Stigma in HIV-positive women. J. Nurs. Scholarsh..

[39] Puigventós F., Riera M., Delibes C., Peñaranda M., de la Fuente L., Boronat A. (2002). Adherence to antiretroviral drug therapy. A systematic review. Med. Clin..

[40] Villa I.C., Vinaccia S. (2006). Adhesión terapéutica y variables psicológicas asociadas en pacientes con diagnóstico de VIH-sida. Psicol. Salud.

[41] Tavera M. (2010). Calidad de vida relacionada a la salud en pacientes con VIH. Rev. Peru. Epidemiol..

[42] Velásquez C. (2011). Results of the implementation of three national guidelines for the prevention of HIV vertical transmission in instituto Nacional Materno Perinatal. Lima, Perú. Rev. Perú. Med. Exp. Salud Publica.

[43] Benito González M.E. (2012). Efectos del ejercicio físico en adultos con VIH/SIDA: Revisión sistemática. Biociencias.

[44] Díaz-Granados C.A., Alvarez C., Prada G., Sarmiento C., Martínez F. (2005). Guía Para El Manejo De VIH/SIDA Basada En La Evidencia.

[45] Higgins J.P.T., Green S. (2011). Cochrane Handbook for Systematic Reviews of Interventions Version 5.1.0.

