# Supplementary material for: Experiences and Attitudes of People with HIV/AIDS: A Systematic Review of Qualitative Studies
Source: Int J Environ Res Public Health. 2020 Jan 19;17(2):639. doi: 10.3390/ijerph17020639 (PMC7014086; doi:10.3390/ijerph17020639)
Supplement: Supplementary file 1 [file ijerph-17-00639-s001.zip › Supplementary-TableS1.docx]

Table S1: Search strategy in the databases

| **DeCS** | **MeSH** | **Entry terms** | |
| --- | --- | --- | --- |
| Virus Inmunodeficiencia  Humana (VIH) | HIV[mh] | Human Immunodeficiency Virus*[tiab]  Human T Cell Lymphotropic Virus Type III[tiab]  Human T Cell Leukemia Virus Type III[tiab]  Lymphadenopathy Associated Virus*[tiab]  Lymphadenopathy-Associated Viruses[tiab]  Human T Lymphotropic Virus Type III[tiab]  AIDS Virus*[tiab]  Acquired Immunodeficiency Syndrome Virus[tiab] | |
|  | **CINAHL/MeSH Subject Headings** |  |  |
|  | Human Immunodeficiency Virus |  |  |
| **Search strategy in PubMed** | | | |
| Search string: | | | Results |
| < (HIV[mj] OR Human Immunodeficiency Virus*[tiab] OR Human T Cell Lymphotropic Virus Type III[tiab] OR Human T Cell Leukemia Virus Type III[tiab] OR Lymphadenopathy Associated Virus*[tiab] OR Lymphadenopathy-Associated Viruses[tiab] OR Human T Lymphotropic Virus Type III[tiab] OR AIDS Virus*[tiab] OR Acquired Immunodeficiency Syndrome Virus[tiab]) AND (qualitative research[mh]) > | | | n= 178 articles |
| **Search strategy in CINAHL** | | |  |
| Search string: | | | Results |
| < (MH "Human Immunodeficiency Virus" OR AB "HIV-1") AND (MH Qualitative studies) > | | | n= 104 articles |
| **Search strategy in Lilacs** | | |  |
| Search string: | | | Results |
| < (HIV OR Virus del sida OR Virus de la inmunodeficiencia humana) AND (cualitativ*) > | | | n= 153 articles |
| **Search strategy in CUIDEN** | | |  |
| Search string: | | | Results |
| < (VIH) AND (CUALITATIVO) > | | | n= 153 articles |
