# Supplementary material for: Experiences and Attitudes of People with HIV/AIDS: A Systematic Review of Qualitative Studies
Source: Int J Environ Res Public Health. 2020 Jan 19;17(2):639. doi: 10.3390/ijerph17020639 (PMC7014086; doi:10.3390/ijerph17020639)
Supplement: Supplementary file 1 [file ijerph-17-00639-s001.zip › Supplementary-TableS2.docx]

| **Table S2:** Results after the methodological evaluation CASPe |
| --- |

| **Source (n=32)** | **1** | **2** | **3** | **4** | **5** | **6** | **7** | **8** | **9** | **Decision** |
| --- | --- | --- | --- | --- | --- | --- | --- | --- | --- | --- |
| Acheampong et al. (2017) | ✔ | ✔ | ❓ | ✔ | ✔ | ✖ | ✔ | ✔ | ✔ | INCLUDED |
| Akhtar et al. (2017) | ✔ | ✔ | ✔ | ✔ | ✔ | ✔ | ✔ | ✔ | ✔ | INCLUDED |
| Biseck, et al. (2015) | ✔ | ✔ | ❓ | ✔ | ✔ | ✖ | ✔ | ❓ | ✔ | INCLUDED |
| Braga et al. (2016) | ✔ | ✔ | ✔ | ❓ | ✔ | ✖ | ✔ | ✔ | ❓ | INCLUDED |
| Carlesso et al. (2011) | ✔ | ✔ | ✔ | ✔ | ✔ | ✔ | ✔ | ✔ | ❓ | INCLUDED |
| Carrasco et al. (2013) | ✔ | ✔ | ❓ | ❓ | ✔ | ✖ | ✔ | ✔ | ✔ | INCLUDED |
| Córdova et al. (2013) | ✔ | ✔ | ✔ | ✔ | ❓ | ✖ | ✔ | ❓ | ❓ | EXCLUDED |
| De la Cruz et al. (2016) | ✔ | ✔ | ✔ | ❓ | ✔ | ✔ | ✔ | ❓ | ✔ | INCLUDED |
| Felisa Muñoz et al. (2012) | ✔ | ✔ | ✔ | ❓ | ✔ | ✖ | ✔ | ❓ | ❓ | EXCLUDED |
| Fernández-Davila et al. (2013) | ✔ | ✔ | ❓ | ✔ | ✔ | ✖ | ✔ | ✔ | ✔ | INCLUDED |
| Figueiredo et al. (2015) | ✔ | ✔ | ✔ | ✔ | ✔ | ✖ | ✔ | ✔ | ❓ | INCLUDED |
| France et al. (2015) | ✔ | ✔ | ✔ | ✔ | ✔ | ✖ | ✔ | ✔ | ❓ | INCLUDED |
| Freitas et al. (2000) | ✔ | ✔ | ❓ | ❓ | ✔ | ✖ | ✔ | ✔ | ✖ | INCLUDED |
| Freitas et al. (2017) | ✔ | ✔ | ❓ | ✔ | ❓ | ✖ | ✔ | ❓ | ✔ | INCLUDED |
| French et al. (2015) | ✔ | ✔ | ❓ | ❓ | ❓ | ✖ | ✔ | ❓ | ✔ | INCLUDED* |
| Gardner (2013) | ✔ | ✔ | ✔ | ✔ | ✔ | ✔ | ✔ | ✔ | ✔ | INCLUDED |
| Gonçalves et al. (2013) | ✔ | ✔ | ✔ | ❓ | ✔ | ✖ | ✔ | ✔ | ✔ | INCLUDED |
| Juarez-Vilchez & Pozo (2010) | ✔ | ✔ | ✔ | ✔ | ❓ | ✖ | ✔ | ✔ | ✔ | INCLUDED |
| Linder et al. (2016) | ✔ | ✔ | ❓ | ✔ | ✔ | ✖ | ✔ | ✔ | ✔ | INCLUDED |
| Lôbo et al. (2012) | ✔ | ✔ | ✔ | ✔ | ✔ | ✖ | ✔ | ✔ | ❓ | INCLUDED |
| Martínez Rojero et al. (2012) | ✔ | ✔ | ✔ | ❓ | ✖ | ✖ | ✔ | ❓ | ❓ | EXCLUDED |
| Matos Oliveira et al. (2016) | ✔ | ✔ | ❓ | ✔ | ❓ | ✖ | ✔ | ✔ | ❓ | INCLUDED |
| Neves & Gir (2006) | ✔ | ✔ | ✔ | ✔ | ✔ | ❓ | ✔ | ❓ | ❓ | INCLUDED |
| Oliveira et al. (2011) | ✔ | ✔ | ✔ | ✖ | ❓ | ✖ | ✔ | ❓ | ❓ | EXCLUDED |
| Oliveira et al. (2015) | ✔ | ✔ | ❓ | ❓ | ✔ | ✖ | ✔ | ✔ | ❓ | INCLUDED |
| Peñarrieta de Córdova et al. (2006) | ✔ | ✔ | ❓ | ✔ | ✔ | ✖ | ❓ | ✔ | ✔ | INCLUDED |
| Pérez Fernández et al. (2008) | ✔ | ✔ | ✔ | ❓ | ❓ | ✖ | ✔ | ✔ | ❓ | EXCLUDED |
| Sikweviva et al. (2014) | ✔ | ✔ | ❓ | ✔ | ✔ | ✔ | ✔ | ✔ | ✔ | INCLUDED |
| Sousa Paiva & Gimeniz Calvão (2004) | ✔ | ✔ | ✔ | ✔ | ✔ | ✖ | ✔ | ❓ | ❓ | INCLUDED |
| Spindola et al. (2015) | ✔ | ✔ | ❓ | ✔ | ✔ | ✖ | ✔ | ✔ | ❓ | INCLUDED |
| Wodajo et al. (2017) | ✔ | ✔ | ❓ | ✔ | ✔ | ✖ | ✔ | ✔ | ✔ | INCLUDED |
| Teixeira et al. (2013) | ✔ | ✔ | ❓ | ❓ | ✔ | ✔ | ✔ | ❓ | ❓ | INCLUDED |
| Note 1:” ✔”= Present; “❓”= Doubtful; “✖”= Not on record | | | | | | | | | | |
| Note 2: We opted to unify the references by mentioning the first and the second author, if there were two authors. From three authors or more, the first is mentioned and we add “et al.” for the rest. | | | | | | | | | | |
| *Even though it does not pass the evaluation criteria, included because of the results’ interest for the object of study.  Note 3: Items (1-9) of the program CASPe to evaluate the articles methodological quality (Cano Arana et al., 2010). | | | | | | | | | | |
| Source: own elaboration | | | | | | | | | | |

**References:**

Acheampong, A.K.; Naab, F.; Kwashie, A. Qualitative exploration of psychological reactions and coping strategies of breastfeeding mothers living with HIV in the Greater Accra Region of Ghana. *Int Breastfeed J* **2017**, 12, 1-8. DOI:10.1186/s13006-017-0119-8

Akhtar, N. F.; Garcha, R. K.; Solomon, P. Experiences of women aging with the human immunodeficiency virus: A qualitative study. *Can J Occup Ther* **2017**, 84(4-5), 253-261. DOI: 10.1177/0008417417722574

Biseck, T.; Kumwenda, S.; Kalulu, K.; Chidziwisano, K.; Kalumbi, L. Exploring fertility decisions among pregnant HIV-positive women on antiretroviral therapy at a health centre in balaka, malawi: A descriptive qualitative. *Malawi Med J* **2015**, 27(4), 128-134.

Braga, R. M. O.; Lima, T. P.; Gomes, A. M. T.; Oliveira, D. C. d.; Spindola, T.; Marques, S. C. Social representations of HIV/AIDS for people living with the syndrome. *Rev enferm UERJ* **2016**; 24(2):e15123; DOI: 10.12957/reuerj.2016.15123

Carlesso, A.; Cecchetto, F. H.; Silva, E. F. d. Women infected by the human immunodeficiency virus: experienced feelings regarding the sickness. *Rev enferm UFPE on line* **2011**, 5(3), 771-777.

Carrasco, P.; Araya Gutiérrez, A.; Loayza Godoy, C.; Ferrer Lagunas, L.; Trujillo Guarda, C.; Fernández Sarmiento, A.; Pérez Cortés, C. How to Understand the Experience of Persons Living with HIV: Implications for Clinical Practice and Research. *Aquichán* **2013**, 13(3), 387-395.

Córdova, F. P.; Luz, A. M. H.; Innocente, A. P.; Silva, E. F. d. HIV seropositive women and their partners facing the decision of a pregnancy. Rev bras enferm **2013**, 66(1), 97-102.

De la Cruz, A.; Caine, V.; Mill, J. Sub-Saharan African immigrants living with HIV in Canada: A narrative inquiry. *International Journal of Migration, Health & Social Care* **2016**, 12(3), 194-210. DOI:10.1108/IJMHSC-12-2014-0046

Felisa Muñoz, S.; Castro, É.; Fiscal Idrobo, L. M.; Narváez Vallejo, L. E.; Paz Cuéllar, J. A.; Villamarín Meneses, M. C. Conocimientos, actitudes y prácticas de las mujeres con VIH durante la gestación y crianza (popayán, 2009). *Inv Enf* **2012**, 14(1), 45-55.

Fernández-Dávila, P.; Morales Carmona, A. Discursos sobre la responsabilidad sexual en hombres VIH-positivos que tienen sexo con hombres. *Rev Esp Salud Publica* **2013**, 87(4), 367-382. DOI: 10.4321/S1135-57272013000400007

Figueiredo, R. M. B.; Thomé, A.; Pinto, P. C.; Prates, C. d. S. Vivências de mães soropositivas para o HIV acompanhadas no serviço de assistência especializada. *Rev Enferm UFSM* **2015**, 5(4), 638-649. DOI: 10.5902/2179769215406

France, N. F.; Mcdonald, S. H.; Conroy, R. R.; Byrne, E.; Mallouris, C.; Hodgson, I.; Larkan, F. N. "An unspoken world of unspoken things": A study identifying and exploring core beliefs underlying self-stigma among people living with HIV and AIDS in Ireland. *Swiss Med Wkly* **2015**, 145, w14113; DOI:10.4414/smw.2015.14113

Freitas, M. R. I.; Gir, E.; Rodrigues, A. R. F. Understanding the sexuality of individuals of HIV-1. *Rev. esc. enferm. USP* **2000**, 34(3), 258-263.

Freitas, M.I.d.F; Bonolo, P. d. F.; Miranda, W. D. d.; Guimarães, M. D. C. Interactions and the antiretroviral therapy adherence among people living with HIV/AIDS. *REME Rev Min Enferm* **2017**, 21(1), e1001. DOI: 10.5935/1415-2762.20170011

French, H.; Greeff, M.; Watson, M. J.; Doak, C. M. HIV stigma and disclosure experiences of people living with HIV in an urban and a rural setting. *AIDS Care* **2015**, 27(8), 1042-1046. DOI:10.1080/09540121.2015.1020747

Gardner, J. The experiences of HIV-positive women living in an African village: perceptions of voluntary counseling and testing programs. *J Transcult Nurs* **2013**, 24(1), 25-32. DOI: 10.1177/1043659612462404

Gonçalves, V. F.; Teixeira, D. Q.; Oliveira, P. F. d.; Sousa, T. H. e. HIV-seropositive women: understanding, feelings and experience before motherhood. *Rev Bras Promoc Saude* **2013**, 26(2), 281-289. DOI: 10.5020/18061230.2013.p281

Juárez-Vílchez, J. P.; Pozo, E. J. Risk sexual behavior among people living with HIV/AIDS and receiving antiretroviral therapy in Piura, Peru. *Rev peru med exp salud publica* **2010**, 27(1), 31-37. Available online: http://www.scielo.org.pe/scielo.php?script=sci_arttext&pid=S1726-46342010000100006&lng=es&nrm=iso (accessed on 10/09/2019)

Linder, V.; Chaves, S. E.; Strapasson, M. Perceptions of living women with human immunodeficiency virus about breastfeeding inability. *Enferm Foco* **2016**, 7(2), 7-11.

Lôbo, M. B.; Silva, S. R. F. F. d; Santos, D. d. S. Bedroom secrets: safe sex knowledge and practice by people living with HIV/AIDS. *Rev Eletr Enf [Internet]* ***2012***, 14(2), 395-403. 10.5216/ree.v14i2.12647.

Martínez Rojero, D.; Rocha Rodríguez, M. d. R.; Herrera Acosta, M. Vivencia de la enfermedad en pacientes con VIH/SIDA en una población zacatecana. *Desarrollo Científ Enferm* **2012**, (20(7), 229-233.

Matos Oliveira, G.; Aguiar Carvalho, M. F. A.; Argolo Teixeira, M.; Cardoso Coelho, E. A.; Teixeira Araújo, R. Perception of seropositive women for hiv about reproductive rights. *J Nurs UFPE on line* **2016**, 10(8), 3028-3033. DOI:10.5205/reuol.9373-82134-1-RV1008201630

Neves, L. A. d. S.; Gir, E. HIV positive mother´s beliefs about mother to child transmission. *Rev. Latino-Am. Enfermagem* **2006**, 14(5), 781-788. DOI: 10.1590/S0104-11692006000500021

Oliveira, A.d.f.; Araújo Vieira, M. C.; Pinheiro Costa e Silva, S.; Mistura, C.; da Silva Jacobi, C.; de Souza Carvalho e Lira, M. O. Effects of HIV in daily life of women living with AIDS. *R pesq: cuid fundam Online* **2015**, 7(1), 1975-1986; DOI: 10.9789/2175-5361.2015.v7i1.1975-1986

Oliveira, D. C. d.; Oliveira, E. G. d.; Gomes, A. M. T.; Teotonio, M. C.; Wolter, R. M. C. P. O significado do HIV/AIDS no processo de envelhecimento. Rev Enferm UERJ **2011**, 19(3), 353-358.

Peñarrieta de Córdova, M. I.; Rivera, A. M.; Piñones Martínez, S.; Quintero Valle, L. M. Experience of living with AIDS in a Latin country: a qualitative analysis. *Cultura de los cuidados* **2006**, X (20), 69-75. DOI: 10.14198/cuid.2006.20.09

Pérez Fernández, P.; Gómez Rodríguez, M. S.; Pinto González, L. Vivencias sentidas hoy por personas que viven con VIH/SIDA. Revista ética de los cuidados 2018, 1(2). Available online: http://www.index-f.com/eticuidado/n2/et6857.php (accessed on 10/09/2019)

Sikweyiya, Y. M.; Jewkes, R.; Dunkle, K. Impact of HIV on and the constructions of masculinities among HIV-positive men in south africa: Implications for secondary prevention programs. *Glob Health Action* **2014**, 7, 24631. DOI: 10.3402/gha.v7.24631

Sousa Paiva, S. d.; Gimeniz Calvão, M. T Feelings of pregnant and post-partum women with HIV/AIDS about not breastfeeding. *Texto contexto - enferm* **2004**, 13(3), 414-419. DOI: 10.1590/S0104-07072004000300011

Spindola, T.; Dantas, K. T. d. B.; Cadavez, N. F. V.; Fonte, V. R. F. d.; Oliveira, D. C. d. Maternity perception by pregnant women living with HIV. *Invest educ enferm* **2015**, 33(3), 440-448. DOI: 10.17533/udea.iee.v33n3a07

Teixeira, S. V. B.; Silva, G. S.; Silva, C. S.; Moura, M. A. V. Women living with hiv: The decision to become pregnant. *R pesq: cuid fundam Online* **2013**, 5(1), 3159-3167; DOI: 10.9789/2175-5361.2013v5n1p3159

Wodajo, B. S.; Thupayagale-Tshweneagae, G.; Akpor, O. A. Stigma and discrimination within the ethiopian health care settings: Views of inpatients living with human immunodeficiency virus and acquired immune deficiency syndrome. *Afr J Prim Health Care Fam Med* **2017**, 9(1), e1-e6. DOI:10.4102/phcfm.v9i1.1314
